# Notes and Notices

**Published:** 1897-02

**Authors:** 


### NOTES AND NOTICES.

### HAHNEMANN.

Brave Hahnemann! Around thy lustrous name
A halo clings. Great healer of mankind,
Traduced by bigots, and by quacks maligned,
Each cycle still unto thy growing fame
Doth add the splendor of a brighter flame.
To thy clear eye and patient student mind
The Herculean labor was assigned:
God's law to read; its message to proclaim.
And well, O faithful servant, brave and true,
Didst thou perform the heavy task imposed;
And well may countless thousands to thee raise
The meed of gratitude and praises due.
The healing streams which thy brave hand disclosed,
Shall keep thy name green in the earth always.

Alton, Mo. OSCAR MACDONALD BROOKS.

DR. EDWARD F. BRADY has removed from Kansas City, Mo., to 3525 St. Louis Avenue, St. Louis, Mo. Dr. Brady has practiced medicine in Kansas City for eleven years. His specialty is mental and nervous diseases, and he finds that St. Louis furnishes a wider field of usefulness.

### HIS KIND OF A PATH.

"There was an old doctor, a-long ago,
Who hired a fellow to shovel his snow,
But instead of a shovel he gave him a hoe,
For he was a hoe-me-a-path, you know."

*L. A. W. Bulletin.*

THE Missouri Institute of Homœopathy, which after the American Institute, is one of the most influential societies in our school, will hold its next annual session in Kansas City, Mo., April 22d, 23d, and 24th, 1897. The present outlook for a large attendance and a profitable session is most encouraging. For more particular information application should be made to Dr. Edward F. Brady, 3525 St. Louis Avenue, St. Louis, Mo.

HAHNEMANN ASSOCIATION OF NEW YORK held its third annual meeting and banquet at Windsor Hotel, on Thursday evening, December 3d, 1896.

President, Francis E. Doughty, M. D.; First Vice-President, Charles McDowell, M. D.; Second Vice-President, Edward Chapin, M. D.; Third Vice-President, D. J. Roberts, M. D.; Recording Secretary, H. D. Schenck, M. D.; Corresponding Secretary, S. H. Vehslage, M. D.; Treasurer, A. G. Warner, M. D.; members of the Executive Committee, J. Lester Keep, M. D., A. B. Norton, M. D., M. Deschere, M. D.

